# Cross-Platform Prediction of Gene Expression Signatures

**DOI:** 10.1371/journal.pone.0079228

**Published:** 2013-11-14

**Authors:** Shu-Hong Lin, Lauren Beane, Dawn Chasse, Kevin W. Zhu, Bernard Mathey-Prevot, Jeffrey T. Chang

**Affiliations:** 1 Graduate School of Biomedical Sciences, University of Texas, Houston, Texas, United States of America; 2 Department of Pharmacology & Cancer Biology, Duke University, Durham, North Carolina, United States of America; 3 Institute for Genome Sciences and Policy, Duke University and Duke University Medical Center, Durham, North Carolina United States of America; 4 Department of Integrative Biology and Pharmacology, University of Texas Health Science Center at Houston, Houston, Texas, United States of America; 5 School of Biomedical Informatics; Institute of Molecular Medicine; Center for Clinical and Translational Sciences, University of Texas Health Science Center at Houston, Houston, Texas, United States of America; National Institute of Genomic Medicine, Mexico

## Abstract

Gene expression signatures can predict the activation of oncogenic pathways and other phenotypes of interest via quantitative models that combine the expression levels of multiple genes. However, as the number of platforms to measure genome-wide gene expression proliferates, there is an increasing need to develop models that can be ported across diverse platforms. Because of the range of technologies that measure gene expression, the resulting signal values can vary greatly. To understand how this variation can affect the prediction of gene expression signatures, we have investigated the ability of gene expression signatures to predict pathway activation across Affymetrix and Illumina microarrays. We hybridized the same RNA samples to both platforms and compared the resultant gene expression readings, as well as the signature predictions. Using a new approach to map probes across platforms, we found that the genes in the signatures from the two platforms were highly similar, and that the predictions they generated were also strongly correlated. This demonstrates that our method can map probes from Affymetrix and Illumina microarrays, and that this mapping can be used to predict gene expression signatures across platforms.

## Introduction

Biological processes are driven by the coordinated actions of multiple gene products. Consequently, the activation of a process, whether it is a cellular activity, activation of a signaling pathway, or other molecular event, is marked by a characteristic change in the expression of a set of genes, which denotes the signature of that process [1]–[3]. In other words, a change in the expression of the genes in a signature is a marker for functional activity. Gene expression signatures developed *in vitro* can be used to predict phenotypes *in vivo* and have been used to predict activation of oncogenic pathways, outcomes in cancers, subtypes of cancers, and sites of cancer metastases [2], [4]–[6].

Gene expression signature analysis is commonly framed as a machine learning problem. First, gene expression data from samples in which a pathway is known to be on or off is collected as a training set. Then, genes that can distinguish these two states are selected and combined into a model that can predict the activation of the pathway in another sample. In our implementation, we select the genes using a Pearson correlation and generate a model using a probit regression, but other approaches have also been explored [7], [8]. An implementation to score activation of oncogenic pathways was recently made available on the web as part of the SIGNATURE project [9].

The power of gene expression signatures is that they combine the expression of multiple genes in a quantitative model. However, as the platforms for measuring gene expression proliferate, the need for approaches to predict signatures across platforms increases. Unfortunately, the technologies for measuring gene expression can be very different and yield gene expression values that are not directly comparable. For example, Affymetrix microarrays measure gene expression using a set of short probes that target a limited region of a single gene [10]. That is, each *probe set* on the Human Genome U133A 2.0 array consists of 11 25-mer probes that target an approximately 400 base pair region on a transcript. The signal values for each probe in a probe set are combined to generate the signal value for the gene. In contrast, Illumina microarrays use a bead-based strategy, where the beads are conjugated to 50 nucleotide gene-specific probes [11]. Because the probe technologies are different, and because the targeted regions may not be the same, the gene expression readings from the two platforms would be expected to vary. Although there are other platforms for gene expression, including those from Agilent or RNA-Seq, for this manuscript, we will focus on Affymetrix and Illumina as they have been arguably the most commonly used to this date.

Previous studies have shown that gene expression measures can be comparable, on the whole, across certain platforms [12], [13]. However, it is not yet known whether higher order comparisons such as those required for signature analysis, which integrates measures across specific groups of genes, are also robust. To address this issue, we have developed signatures using both the Affymetrix and Illumina microarrays, two commonly used platforms, and compared the performance of methods to interconvert gene expression measures. We find that the signatures from the two platforms use very similar sets of genes, and that their predicted pathway activities are highly comparable. Taken together, these analyses provide a proof-of-concept and blueprint for developing the capacity to navigate the upcoming world of multi-platform gene expression measures.

## Results

### Concordance of Gene Expression across Affymetrix and Illumina Microarrays

**Figure 1:**
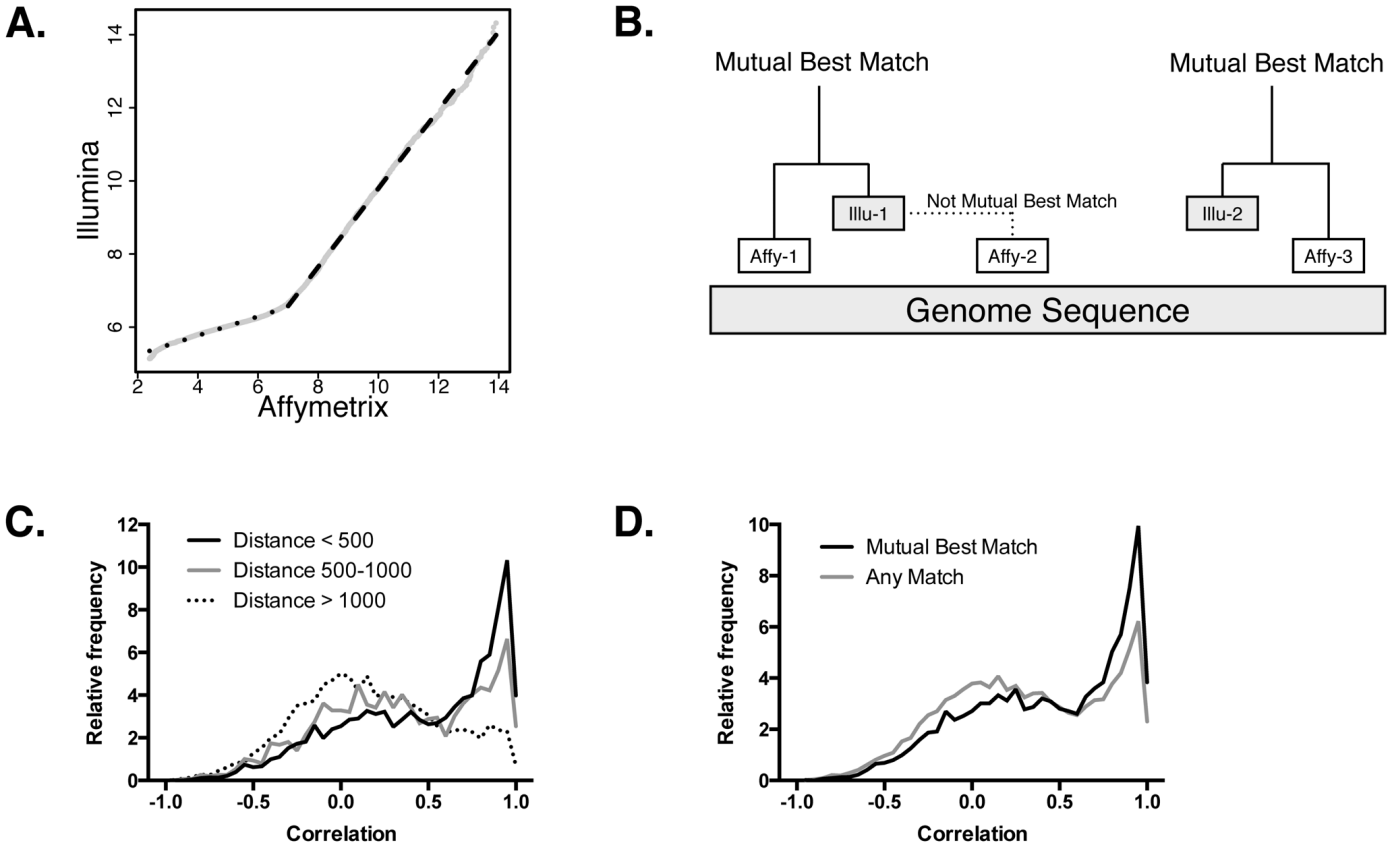
Correspondence of gene expression between the Affymetrix and Illumina platforms. A. The signal values on Affymetrix and Illumina microarrays are bilinear. The x-axis shows the distribution of gene expression values of a melanoma tumor on an Affymetrix microarray. The y-axis shows the distribution of the expression of the same sample on an Illumina microarray. Both high and low expression values are linearly related, but with different slopes. B. Probes from Affymetrix and Illumina microarrays are mapped onto their target in the human genome. The distance to the nearest probe of the other platform are calculated. If a pair of probes from the opposing platform are both closest to each other, they are considered *mutual best matches*, for example, *Affy-1* and *Illu-1*. However, *Illu-1* and *Affy-2* are not mutual best matches. While *Illu-1* is the best match for *Affy-2*, the converse is not true in this case. C. This histogram shows the correlation of the expression profiles of probes that are different distances apart. There is an enrichment of correlations close to 1.0 in probes that are closer. Probes that are over 1000 base pairs apart show no enrichment of highly correlated probes. D. This shows the differences in the correlations between pairs of probes that are mutual best matches and ones that are not. Probes that are mutual best matches have higher correlations than otherwise.

We collected total RNA from 16 melanoma tumors and hybridized aliquots to Affymetrix HG-U133A 2.0 (AFFY) and Illumina Human HT-12 v4.0 (ILLU) arrays. Then, we compared the distribution of the gene expression signal values for each sample and found a pattern that resembles a bi-linear distribution (Figure 1A). At high expression values (signal greater than 7 in AFFY and 6.7 in ILLU; roughly 46% of the probes), the AFFY signal increased by 1.07 for every unit increase in ILLU. This relationship indicated that the relative differences in the expression levels of highly expressed genes were roughly equal. This suggested that a linear model could be applied across platforms, although the model may be different depending on the expression level of the gene.

Next, to evaluate the concordance of the expression patterns of individual genes, we compared strategies to match corresponding probes across platforms. For each AFFY probe set or ILLU probe, we mapped the targeted region to the human genome (Figure 1B). For the AFFY probe, we found the closest ILLU probe on the same gene, and repeated the complementary procedure with the ILLU probes. That is, for each probe, we identified the closest from the other platform. Because each platform could have multiple probes for the same gene, two AFFY probes may share the same closest ILLU probe. However, the ILLU probe will be closest to only one of those AFFY probes (unless they are equidistant). We called pairs that are both closest to each other *mutually best matches.* We further annotated these probe mappings by calculating their distance across the genome (number of bases).

The goal of the probe matching was to identify sets of probes that were likely to have similar expression patterns across platforms. To evaluate this, we calculated the Pearson correlation of the corresponding probes across the melanoma samples. First, to determine the impact of the distance between corresponding probes, we grouped the matches according to distance, <500 base pairs (bp) apart, 500–1000 bp, and >1000 bp. Among the matches, 9,178 pairs were less than 500 bp apart, while 1,492 and 9,784 probes were 500–1000 bp and >1000 bp apart, respectively. As shown in Figure 1C, the correlation of the expression of the probe pairs varied depending on their distance (*p*<6.6×10^−16^; Kruskal-Wallis rank sum test). Furthermore, probes that were mutually best matches were more correlated than best matches that were not mutual (Figure 1D; *p*<2.2×10^−16^; Mann-Whitney U test). These results showed that a subset of the probes had higher correlation, and consequently, to accurately convert expression profiles, we can use these criteria to focus on the probes that are most likely to be concordant.

### Similarity of Genes in Gene Expression Signatures

**Figure 2:**
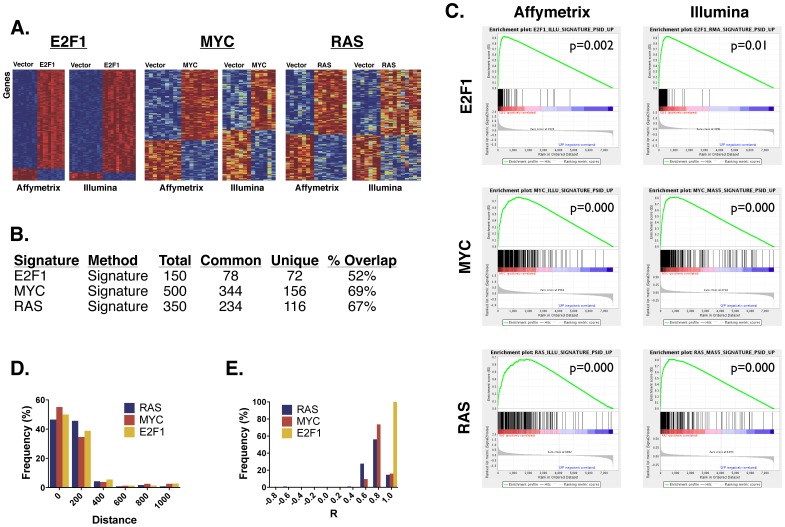
Comparison of gene expression signatures across the Affymetrix and Illumina platforms. A. These heatmaps show the gene expression signatures of the E2F1, MYC, and RAS pathways collected on Affymetrix and Illumina. Each row in the heatmap represents a gene in a signature. Each signature contains a different set of genes, so the genes are not aligned across all heatmaps. Each column represents a sample expressing either a vector control or pathway gene. The color indicates the expression level of the gene, where red indicates high expression, and blue indicates low expression. B. This table shows the number of genes in each signature. *Total* indicates the total number of genes in the signature, *Common* is the number of genes shared between the Affymetrix and Illumina platforms, *Unique* is the number of genes unique to each platform, and *% Overlap* is the percent of genes shared. C. These are GSEA Enrichment plots that show the similarity between the genes in the Affymetrix and Illumina signatures. Each of the three rows contains a different signature. The Affymetrix signatures are in the left column, and the Illumina ones are on the right. In the top left plot, the Affymetrix gene expression file are sorted from left to right according to the GSEA signal-to-noise metric, where the ones most highly associated with E2F1 are on the left, and the ones most highly associated with the vector control are on the right. The vertical black bars in the middle of the plot show the position of the genes associated with E2F1 in the Illumina signature. Most of the black bars are clustered on the left, indicating a high concordance of the genes associated with E2F1 across the two platforms. The other plots can be interpreted similarly. D. This histogram shows the relative percent frequency of the genomic distance between the Affymetrix and Illumina probes that are shared in the RAS, MYC, and E2F1 signatures. E. This histogram shows the relative percent frequency of the Pearson correlation of the expression values between the probes in the Affymetrix and Illumina signatures.

While the previous analysis showed that gene expression patterns could be, overall, concordant between the AFFY and ILLU platforms, we next examined the similarity of the genes that comprised gene expression signatures. For this analysis, we collected ∼10 biological replicates of human mammary epithelial cells expressing either a GFP control, E2F1, MYC, or RAS. We hybridized total RNA from those samples onto both AFFY and ILLU arrays. Then, we mapped the probes across the arrays by selecting the ones that were mutual best matches within 1000 base pairs, the subset of probes that were previously found to exhibit highest correlation in expression. Finally, we generated gene expression signatures on both platforms using the CreateSignature tool [9]. Using parameters previously established [4], we used 150, 500, and 350 genes for the E2F1, MYC, and RAS signatures, respectively. On both platforms, we were able to select genes that could differentiate the controls from the other samples (Figure 2A). By visual examination of the heat maps, we noted that the samples for the MYC and RAS signatures on ILLU exhibited greater heterogeneity in gene expression than could be seen on AFFY. However, we could not distinguish whether this was due to an intrinsic difference in the platforms, or whether it was due to other technical reasons (see Methods).

Comparing the genes selected, we found that 52% to 69% of the genes were shared in the signatures between the two platforms (Figure 2B). Although this was already a highly significant overlap, we examined whether the genes from the AFFY signature that did not meet the cutoff for the ILLU signature still had a strong score (Figure 2C). To do this, we scored the AFFY genes in the ILLU data set using GSEA [14] and found that they were enriched (*p* = 0.01). The converse comparison was also significant (*p* = 0.002). Similar results were seen for the MYC and RAS signatures.

Next, we assessed the quality of the common genes. Using the genes that occur in the signatures for both platforms, we looked at the distance between the probes across the platforms (Figure 2D). Since we had restricted our selection to a set of probes that occurred within 1000 base pairs or less of each other, the maximum distance was 1000. However, we saw that the vast majority of probes were found within 400 base pairs of each other. The MYC signature had the most probes within 200 base pairs, while RAS had the least. In addition to this distance, we also examined the Pearson correlation of these common probes in the gene expression data across the two platforms (Figure 2E). Nearly all probes had a correlation of at least R = 0.4, while all of the E2F1 probes are correlated at R>0.80. Of the three signatures, RAS had the least correlated probes.

Taken together, the above results showed a large degree of similarity in the genes that comprised the signatures across platforms, as well as high concordance in the expression values of these genes. Although the exact identities of the genes were not the same, their patterns of expression were preserved on the whole.

### Comparing Predictions from Gene Expression Signatures

**Figure 3:**
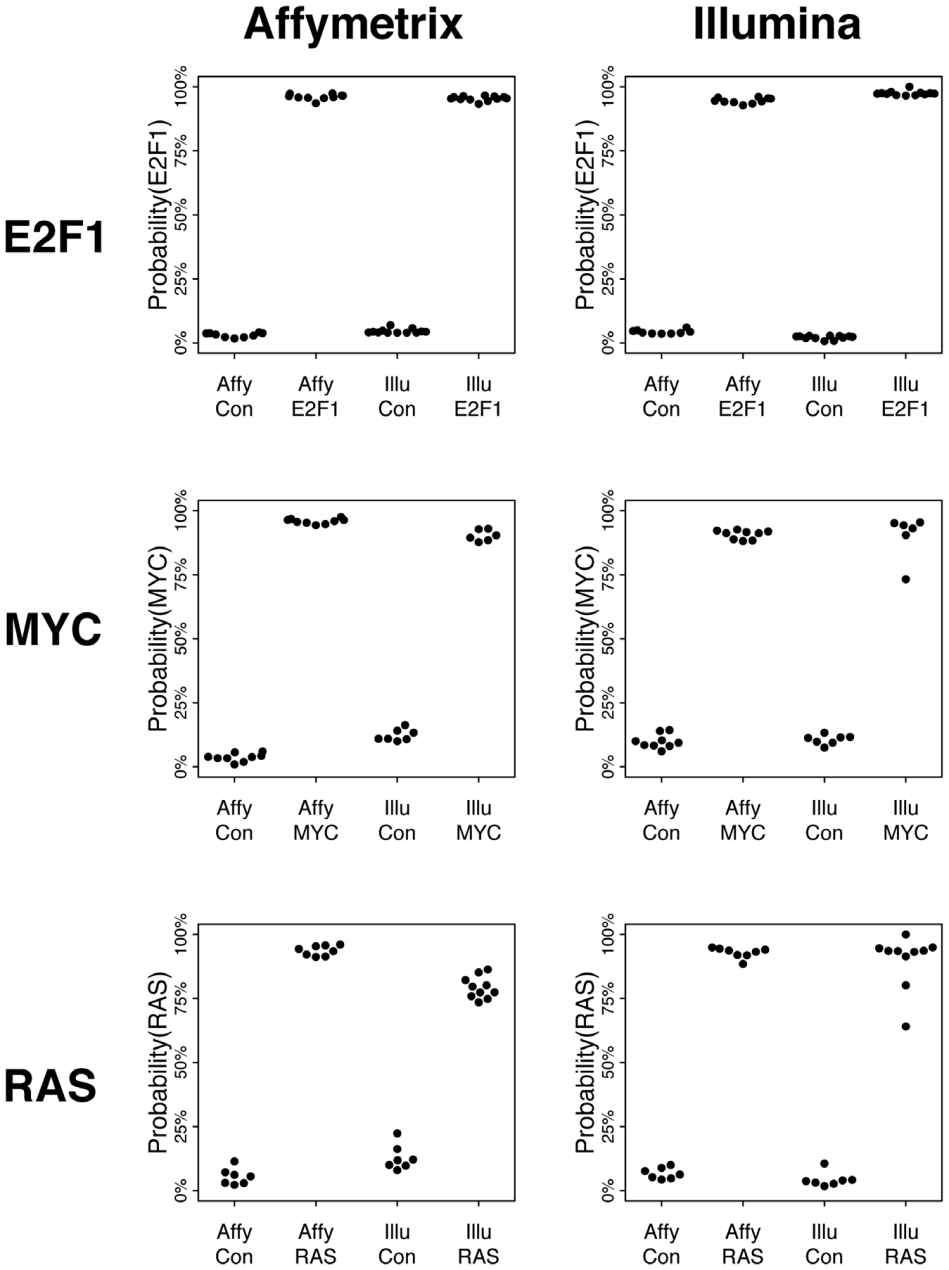
These plots show the ability of the Affymetrix and Illumina signatures to predict the activation of pathways. Each of the three rows contains a different signature. The Affymetrix signatures are in the left column, and the Illumina ones are on the right. On the top-left plot, the y-axis shows the predicted probability of the E2F1 pathway. The x-axis is comprised of four groups of predictions. The first group contains the predicted probabilities of the Affymetrix E2F1 signature on the Affymetrix control samples (calculated using a cross-validation strategy). The second are the probabilities of this signature on the Affymetrix E2F1 samples. The third and fourth are the probabilities of this signature on the Illumina control and E2F1 signatures. The remaining five plots can be interpreted similarly.

Finally, we examined whether we could apply the signatures to predict the activation of pathways across platforms. Using the E2F1 samples on AFFY as the training set, we applied CreateSignature to predict their score on the AFFY and ILLU samples (with leave-one-out cross-validation). We found that across platforms, the control samples (with E2F1 off) received low probabilities while the E2F1-expressing samples (with E2F1 on) had high scores (Figure 3). We saw the same pattern using the ILLU samples as the training set. The MYC and RAS signatures also showed a strong distinction between the control and pathway activated samples, although the distinctions in the ILLU scores were slightly lower than could be seen in E2F1. The probabilities in the control samples were slightly higher, and the ones in the pathway-activated samples were slightly lower. However, there was still a strong and clear distinction between the two types of samples.

**Figure 4:**
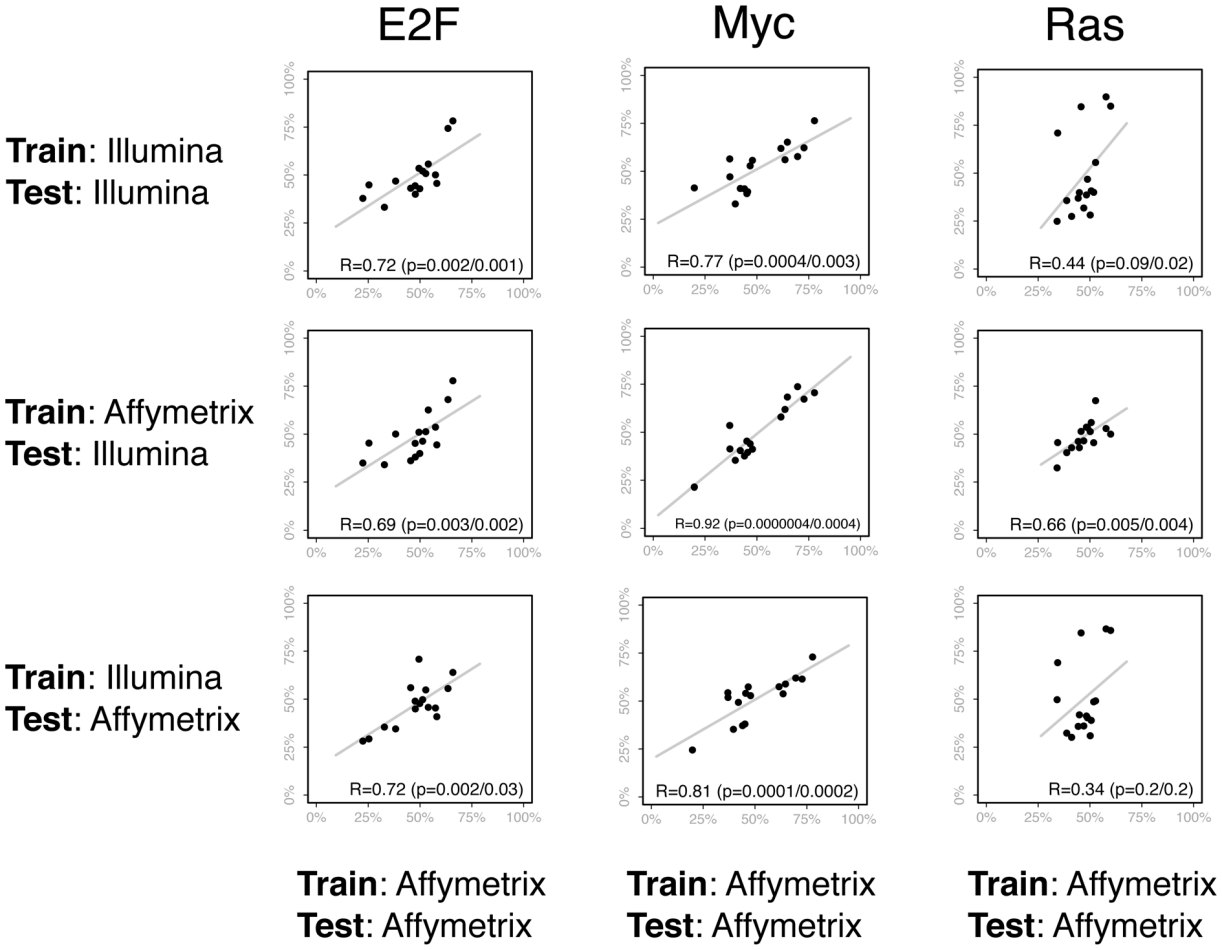
These plots compare the predictions of the E2F, Myc, and Ras pathway signatures when different training and test sets are used. In the scatter plots, each of the points represents one of 16 melanoma tumor samples. On the x-axis is the prediction of the pathway signature when the Affymetrix data is used for both the training and test set. On the y-axis (for the first row) are the predictions when Illumina data is used for both the training and test set. Thus, the first row shows a comparison between the predictions made from Affymetrix or Illumina arrays. For the second row, the y-axis contains the predictions made across platforms, when Affymetrix is used for the training set and Illumina is used for the test. In contrast, the third row contains the predictions when Illumina is used for the training set and Affymetrix for the test. The Pearson correlations and regression lines are also shown in each plot. Two *p*-values are reported. The first is from a Pearson’s *r*, and the other uses a non-parametric Spearman’s rank correlation coefficient to account for predictions that are non-linear.

To compare the predictions of signatures across platforms, we applied the E2F, MYC, and RAS signatures to the melanoma data set. As a baseline, we used the AFFY data for both the signatures and the melanoma data set. Then, we compared these to three cross-platform conditions: 1) both the training data (the three pathways) and the test data (the melanoma data set) were on the ILLU platform, 2) the training data was on AFFY and the test was on ILLU, and 3) training AFFY and test ILLU (Figure 4).

Comparing the cross-platform predictions, all showed significant similarity to the baseline, with one exception. The predictions for the RAS signature were most variable, with several outliers seen in the predictions made in the Train:Illumina/Test:Illumina and Train:Illumina/Test:Affymetrix conditions, although the direction of the predictions were generally correct. This parallels the observation that the probes that comprise the RAS signature were the least concordant (with respect to distance and expression values) of the three signatures. In all other comparisons, there was a clear linear relationship between the cross-platform predictions and the predictions made entirely on the Affymetrix platform. These results showed that the Illumina platform could generate similar signature predictions as the Affymetrix platform, that they were similar even if the training and test data were on different platforms, and that we have a probe mapping strategy that could reliably reproduce signature predictions.

## Discussion

As the number of popular platforms to measure gene expression increases, the ability to compare and integrate gene expression measures across diverse platforms becomes correspondingly important. Prior studies have shown an overall concordance of gene expression measures across platforms, and in this work, we have extended those studies to show that predictions from gene expression signatures can also be robust across the Affymetrix and Illumina microarray platforms. One critical issue with gene expression platforms that we have not addressed is whether signatures or gene sets are portable across single channel array platforms (such as the ones we analyzed) and dual channel ones (e.g. Agilent). The degree of difficulty in converting signals with that include different channels of information remains unknown.

Some principles emerge from our analyses. The first is that expression measurements between probes targeting similar regions of the gene are most robust. This may be due to biological (distant probes are more likely to probe distinct isoforms) or technical (close probes may have similar hybridization characteristics or suffer from similar degradation effects) reasons. Secondly, the similarity between the gene expression signature predictions between the Affymetrix and Illumina platforms shows that relatively simple mapping procedures are robust enough to preserve the changes in transcriptional profiles that best capture biological phenotypes.

We believe that our approach provides a model for future analyses comparing gene expression signatures across platforms. As new technologies for gene expression measurements are developed, for example sequencing-based approaches [15], the need to integrate data across platforms will intensify. Having an understanding of the capacity to convert measures across platforms will be necessary to derive the full value across the entire corpus of transcription profiles that have been generated.

## Materials and Methods

### Data Sets

To generate the Myc and Ras gene expression signatures, we used total RNA that was previously collected from a prior project [2]. Although total RNA for E2F was also collected in that project, no more remained. Therefore, we re-generated RNA for E2F using the same procedure. We cultured HMEC in low serum medium for 36 hours, and then transduced them with adenoviruses expressing either E2F1 or GFP at a multiplicity of infection of 150. 16 hours after transduction, we collected total RNA using Qiagen RNeasy Mini Kits. For the 16 melanoma samples, we used total RNA collected in a prior project [16]. We processed the RNA for the E2F and melanoma samples on both Affymetrix HG-U133Av2 and Illumina HumanHT-12 v4 microarrays. The newly generated Affymetrix and Illumina data were deposited into the Gene Expression Omnibus under accession GSE50051. For the Myc and Ras samples, we used the Affymetrix data generated previously from those samples and processed them on Illumina only.

Affymetrix CEL files were preprocessed with RMA and MAS5 using Bioconductor [17], [18]. Illumina IDAT files were preprocessed using the IlluminaExpressionFileCreator module on GenePattern [19]. The Affymetrix data sets contained 22,277 probe sets, and the Illumina data sets contained 47,323 probes.

### Matching Probes

To find corresponding probes between the Affymetrix and Illumina platforms, we aligned each of the probes to the human genome sequence. For the Affymetrix probe sets, we extracted the targeted region using files available from the NetAffx resource on the Affymetrix website. The probe sequences for the Illumina probes were available from the Illumina website. We mapped both the Affymetrix and Illumina probes to human genome assembly hg18 from the UCSC genome database using the BLAT algorithm [20], [21]. This generated for each probe the genomic coordinate of the region that was targeted. We calculated the distances between every pair of probes as the number of bases between the centers of each probe. For each Affymetrix probe, we found the closest Illumina probe on the same gene. We performed the converse operation for the Illumina probes. If a pair of Affymetrix and Illumina probes were closest to each other, we considered them *mutually best matches*. To merge an Affymetrix and Illumina data set, we aligned the probes that were mutually best matches and discarded all non-matching probes. This yielded merged data sets with 7,589 probes.

### Signature Predictions

We used the CreateSignature module on the GenePattern instance running at http://genepattern.uth.tmc.edu/ [9]. For the signature analyses, we used parameters previously established [4]. For E2F1, we used the RMA preprocessed data with 150 genes, 2 metagenes, and quantile normalization. For Ras, we used the MAS5 preprocessed data with 500 genes, 2 metagenes, and quantile normalization. For MYC, we used the MAS5 preprocessed data with 350 genes, 3 metagenes, and quantile normalization.
